# Phytochemical profile and biological activities of *Caesalpinia coriaria* extract: a review

**DOI:** 10.3389/fvets.2025.1629447

**Published:** 2025-07-22

**Authors:** Moises Cipriano-Salazar, Mohamed Z. M. Salem, Mona M. M. Y. Elghandour, Shady Selim, Maximilian Lackner, Abdelfattah Z. M. Salem

**Affiliations:** ^1^Facultad de Medicina Veterinaria y Zootecnia No. 1, Universidad Autónoma de Guerrero, Chilpancingo, Mexico; ^2^Forestry and Wood Technology Department, Faculty of Agriculture (El-Shatby), Alexandria University, Alexandria, Egypt; ^3^Facultad de Medicina Veterinaria y Zootecnia, Universidad Autónoma del Estado de México, Toluca, Mexico; ^4^Department of Pesticide Chemistry and Technology, Faculty of Desert and Environmental Agriculture, Matrouh University, Mersa Matruh, Egypt; ^5^Department of Industrial Engineering, University of Applied Sciences Technikum Wien, Vienna, Austria; ^6^Dipartimento di Scienze del Suolo, Della Pianta e Degli Alimenti, Università Degli Studi di Bari, Bari, Italy

**Keywords:** *Caesalpinia coriaria*, bioactive compounds, antimicrobial activity, antiparasitic activity, plant extracts, polyphenols

## Abstract

*Caesalpinia coriaria* (Jacq.) Willd [syn.: *Libidibia coriaria* (Jacq.) Schltdl.], a member of the Fabaceae family and the Caesalpinioideae subfamily, is commonly known in Mexican vernacular as “cascalote“. Various botanical parts of this tree, such as leaves, pods, flowers, seeds, branches, and bark, have been studied due to their bioactivity and their astringent, antiparasitic, antiseptic, and anti-inflammatory properties. Extracts obtained from *C. coriaria* contain a wide range of bioactive compounds, including tannins, terpenoids, phenols, coumarins, quinones, flavonoids, saponins, carbohydrates, proteins, glycosides, cardiac glycosides, anthraquinones, steroids, and polyphenols. During the fattening phase in ruminants, these plant extracts may be used to reduce gastrointestinal parasitism, promote growth, and decrease drug residues in animal-derived products. This review aims to highlight the importance of the bioactivities of *C. coriaria* extracts and their active compounds. *In vitro* studies have demonstrated that the phenolic and flavonoid compounds present in this species inhibit bacterial growth by disrupting membrane integrity and enzymatic activity, often outperforming conventional antibiotics. In livestock production systems, the presence of pathogenic bacteria leads to significant economic losses; in this context, the use of polyphenolic compounds derived from *C. coriaria* may have a positive effect on animal productivity. Moreover, the extracts from this tree represent a promising source of bioactive compounds for various industrial applications.

## Introduction

In recent years, the use of plant-derived extracts, particularly from legumes and aromatic species, has generated growing interest as a natural and ecological alternative to synthetic antibiotics and anthelmintics in animal production systems (1). This trend reflects the need to improve animal health and productivity while mitigating the risks associated with antimicrobial resistance, pharmaceutical residues in animal products, and the environmental impact of livestock farming (1, 2). In this context, *Caesalpinia coriaria* (Jack) Willd. (syn.: *Libidibia coriaria* (Jacq.) Schltdl.), commonly known in Mexico as “cascalote,” is a species native to tropical and subtropical regions that belongs to the Fabaceae family (3); however, despite being a legume, it does not have the capacity to fix atmospheric nitrogen and therefore does not contribute to enriching the soil with this element (4). Its presence predominates on the Pacific coasts in the states of Oaxaca, Michoacán, Jalisco, and Sinaloa (4, 5) and is known to be a species commonly used by traditional medicine for the treatment of various ailments.

**Figure 1:**
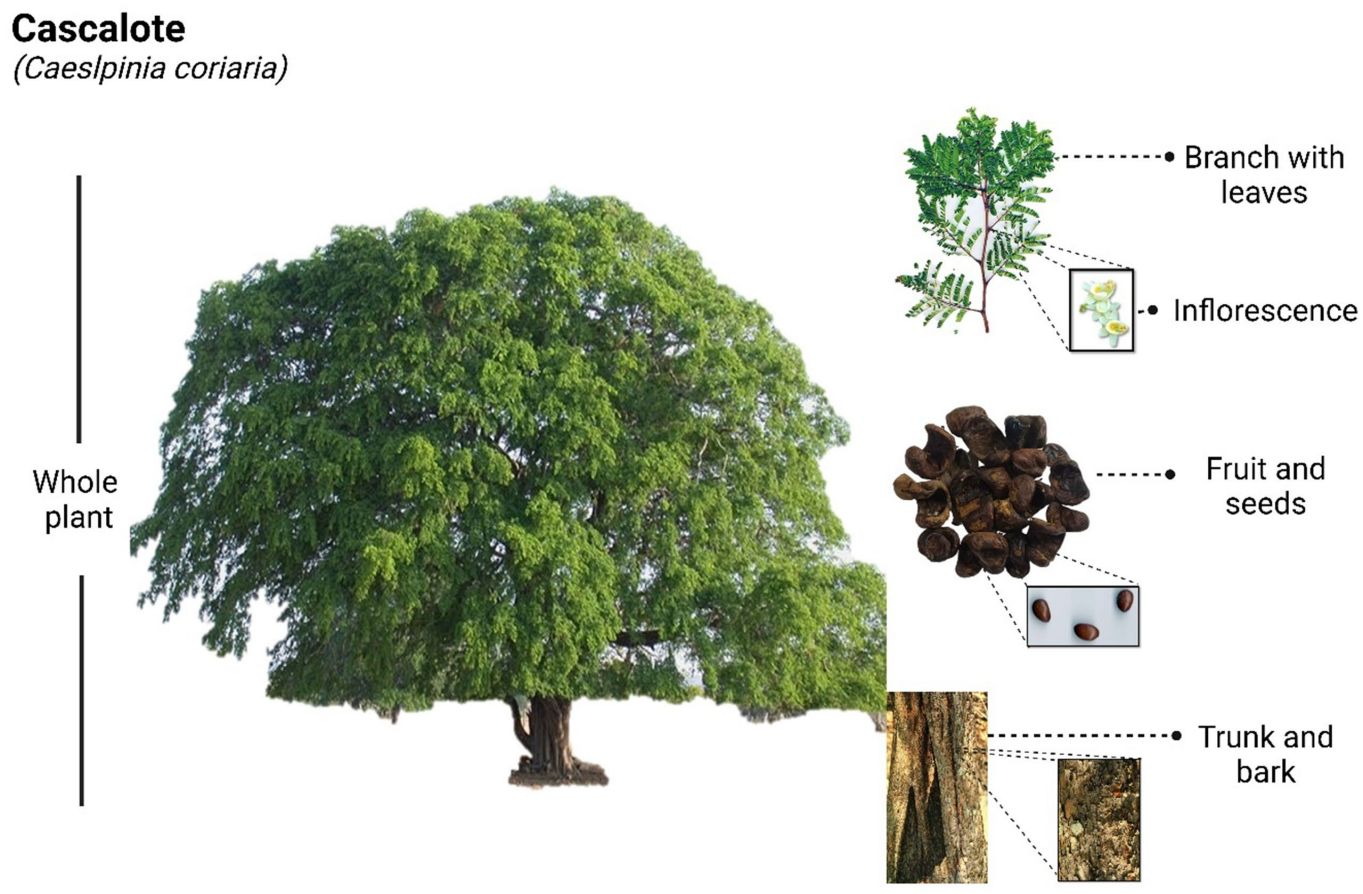
*Caesalpinia coriaria* tree with its botanical parts.

*Caesalpinia coriaria*, which can grow up to 6 meters tall, bears individual thorns along its branches and produces orange or yellow flowers with red stripes, measuring up to 1.5 cm in diameter. Its green leaves, with pale undersides, are composed of three to five pairs of oval leaflets (2 to 6 cm long), and its leguminous fruits are small, reddish, cylindrical pods that typically contain about five seeds each (Figure 1). All parts of the plant are used to treat various ailments, including the bark and leaves as astringents, the flowers for heart disease and digestive issues, the roots for their antiseptic properties in ulcer treatment, and nut-based infusions for relieving tonsillitis (6). The applications of this species extend beyond human medicine, as several studies have demonstrated its potential as ruminant fodder with promising results, and its use in artisanal leather tanning processes is also well docum (4). Incorporating *C. coriaria* fruit waste into ruminant diets reduces methane and carbon dioxide emissions while improving ruminal fermentation, offering an eco-friendly approach to livestock management (7, 8). The combined use of *C. coriaria* fruit and fungal agents provides an effective, sustainable alternative for controlling gastrointestinal nematodes in sheep (9). Its fruit, which resembles a twisted pod, is called *nacascolotl* in Náhuatl, meaning “twisted ear,” and is the most valued part of the plant due to its astringent, antiseptic, and anti-inflammatory properties (10).

*Caesalpinia coriaria* is a leguminous tree with well-documented ethnomedical applications (11, 12), whose pharmacological relevance lies in its content of bioactive phenolic compounds with anthelmintic properties, particularly effective for controlling parasitic infections in cattle and small ruminants (13–15); this makes it a valuable resource for farmers who lack access to synthetic veterinary drugs, as the use of its extracts during the fattening phase not only contributes to reducing gastrointestinal parasitism, but also enhances growth performance and minimizes drug residues in animal-derived products (16). The therapeutic potential of this species is further supported by the long-standing use of its leaves and fruits for their anti-inflammatory (10), antioxidant (17), and antibacterial (18, 19) activities, with traditional preparations such as decoctions of dried fruits and leaves being employed to relieve gastrointestinal discomfort and stomach cramps (20). In particular, the pods are known to contain high concentrations of phenolic compounds with strong antioxidant capacity (21, 22), while both leaves and fruits have been found to be rich in saponins, tannins, flavonoids, ethyl gallate, and gallic acid (15, 18, 22), all of which contribute to their bioactivity. Among these, methyl gallate, ethyl gallate, and corilagin stand out as predominant phenolic constituents, whose multiple biological activities, including potent free radical scavenging effects, have been identified and described in several studies (3, 21, 23–25).

By modulating nitric oxide production, as well as anti-inflammatory and antioxidant pathways, methanol extracts of *C. coriaria* pods significantly reduced gastrointestinal lesions in rat models, showing effects comparable to conventional medications, possibly due to the presence of gallic acid derivatives (21). The hydroalcoholic extracts from fruits exhibit larvicidal and ovicidal properties against the parasitic worm (*Haemonchus contortus* Rudolphi, 1803) Cobb, 1898 (family Trichostrongylidae) in ruminants, indicating that they may be used as a natural anthelmintic (9, 26). To emphasize the biological benefits of *C. coriaria* extracts from various botanical parts and their bioactive components, this review was conducted.

### Extraction techniques for phytochemical analysis

In the field of medicinal and aromatic plants, there are several methods for the extraction from different parts of plants (seeds, leaves, bark, wood, roots, flowers, and branches) including soaking or maceration, solid–liquid extraction, and others using several solvents (27–31). In this regard, *C. coriaria* fruits were extracted using maceration methods with two solvents: polar (ethyl acetate) and less polar (hexane) solvents (32). To generate the hydroalcoholic extract, which was then concentrated using a rotary evaporator at 50–55°C, the dried fruit material was macerated with water and methanol (70%, 1:10, w/v) or aqueous methanol at room temperature for 24 h (13, 15, 24). An organic fraction; ethyl acetate (EtOAc-F) and an aqueous fraction; water (Aq-F) were obtained by liquid–liquid extraction of the hydroalcoholic extract.

At room temperature, 600 g of the collected pods were dried, ground into a powder, and extracted using 2 L of a solvent mixture of ethanol, acetone, and water (80:10:10, v/v). The extract after 72 h was filtered and concentrated under lower pressure (3). In triplicate, 900 g of *C. coriaria* pods were macerated in 2 L of methanol for 24 h at room temperature. The Whatman filter paper was used to filter the resultant extract. A rotary evaporator was used to remove the solvent following filtering (21). Dried *C. coriaria* fruits were ground to 1 mm using a hammer mill to create the aqueous extract. In 2.5 L of distilled water, 1 kilogram of pulverized *C. coriaria* fruits were steeped. The contents were filtered to produce the aqueous extract after the combination was allowed to stand for 72 h (16). Additionally, the chromatographic analysis methods for identifying chemical compounds from *C. coriaria* were primarily employed in high-pressure liquid chromatography (HPLC) and gas chromatography–mass spectroscopy (GC–MS) analyses (32).

### Phytochemical profile and bioactive compounds

**Figure 2:**
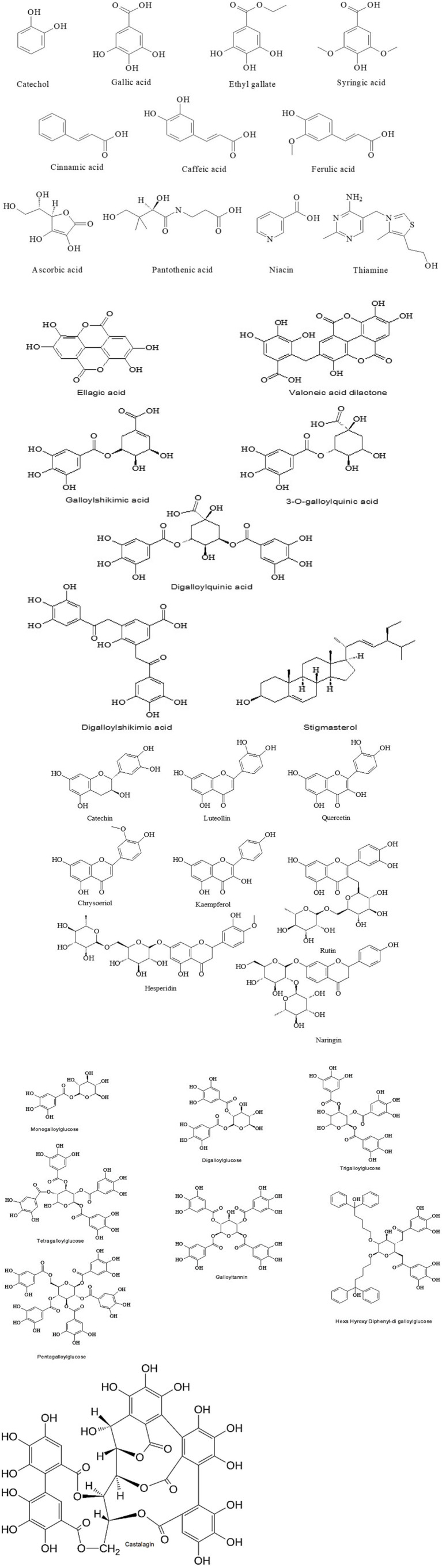
Chemical structures of the compounds present in *Caesalpinia coriaria* extracts (15, 16, 21, 24, 32).

Figure 2 displays the chemical structures of the bioactive substances found in *C. coriaria* extracts as collected from the literature, and Table 1 shows the fruits’ proximate analysis. The extract of *C. coriaria* has been reported to contain phenols, tannins (including condensed tannins and proanthocyanidins), flavonoids, quinones, coumarins, and saponins (25, 33). Tannins are polyphenolic secondary metabolites derived from gallic acid, characterized by their high molecular weight, water solubility, and bitter taste (8); they are synthesized by plants during their growth and development and are distinguished by their ability to form stable, high-strength complexes with proteins (4, 34). Based on their chemical structure, tannins are broadly classified into two main types: hydrolyzable and condensed tannins. In the methanolic extract of *C. coriaria* pods, considerable concentrations of total polyphenolic compounds (439.08 mg/g), condensed tannins (7.72 mg/g), flavones and flavonols (149.50 mg/g), as well as total flavonoids (16.84 mg/g) have been reported (21). One of the main reasons *C. coriaria* has attracted significant research interest is its high tannin content, particularly concentrated in the leaves and pods (5). Furthermore, ethyl acetate and hexane extracts obtained from the fruits have shown insecticidal activity against *Spodoptera frugiperda* (32). HPLC analysis identified phenolic compounds, including ellagic acid, in the ethyl acetate extract, while GC–MS analysis of the hexane extract revealed hexadecanoic acid, 11-methylheptacosane, dodecanoic acid, and nonacosane as the major constituents (32).

**Table 1:** Proximate analysis of *Caesalpinia coriaria* fruits^*^.

Component	Quantity (%)
Humidity	3.0
Dry matter	97.0
Ash	2.43
Crude protein	4.84
Ether extract	0.19
Crude fiber	3.35
Neutral detergent fiber	10.30
Acid detergent fiber	8.18
Nitrogen free extract	83.50
Hydrolyzable tannins	35.5
Condensed tannins	10.4

^*^Data from (4, 36, 88).

Given that the phenolic compounds present in the pods are recognized for their antioxidant properties, the concentration of these compounds in the methanolic extract of *Caesalpinia coriaria* pods is likely to influence the plant’s overall antioxidant capacity. According to liquid chromatography-mass spectroscopy (LC–MS) analysis, the main constituents of the extract include gallic acid, 3-O-galloylquinic acid, digalloylglucose, tetragalloylglucose, valoneic acid dilactone, pentagalloylglucose, digalloylshikimic acid, and ellagic acid (Figure 2; 21). These compounds have demonstrated not only strong antioxidant activity but also significant efficacy in reducing methane emissions when incorporated into animal diets, along with antibacterial (35) and antiparasitic properties. Estimates suggest that the tannin content in *C. coriaria* fruits ranges from 34 to 47% (36), with some studies reporting approximately 35% hydrolyzable tannins and around 10% condensed tannins (4), and it is further estimated that approximately 20,000 tons of *C. coriaria* pods are produced annually in Mexico (5), with tannin extraction from the fruit powder yielding up to 47.0% by weight in total tannins, of which 30.0% corresponds to hydrolyzable tannins (37).

Ethyl gallate and gallic acid were identified and characterized through spectroscopic data analysis and comparison with previously published literature, while stigmasterol was confirmed by direct comparison with the spectroscopic data of a reference standard (3, 5, 38). Gallic acid has been described as the main compound in a hydroalcoholic extract of *C. coriaria* (15, 24, 39), and its fruit extract has also been shown to contain water-soluble vitamins such as thiamine, pantothenic acid, and niacin (16). Quantitative and phytochemical analyses of the pod material showed that the tannin fraction is what gives it its antibacterial properties. According to the findings, *C. coriaria* may be a good choice for managing organisms with antibacterial properties (19).

### Antimicrobial activity

Extracts and isolated compounds from *C. coriaria* exhibit significant antibacterial effects against human pathogens (e.g., *Escherichia coli*, *Staphylococcus aureus*, *Pseudomonas aeruginosa*, *Salmonella typhi*, and *Listeria monocytogenes*) and aquaculture-relevant bacteria (*Aeromonas* spp.), often outperforming standard antibiotics *in vitro* (7, 24, 40). The bioactive substances flavonoids and glycosides from the ethanolic extract of *C. coriaria* penetrate the inner membrane and deactivate the respiratory chain dehydrogenase enzyme system of *E. coli*, *S. aureus*, and *Klebsiella pneumonia*, preventing cell growth and respiration (41). Table 2 summarizes the antimicrobial potential.

**Table 2:** Antimicrobial activity of extract/bioactive compounds from *Caesalpinia coriaria*.

Extract/the isolated compounds	Plant part	Antibacterial effects	References
Hydroalcoholic extract	Fruits	Antibacterial action against *E. coli*, *P. aeruginosa*, *S. typhi*, *L. monocytogenes*, and *S. aureus*.	(24)
Methyl gallate and gallic acid	Ethyl acetate fraction from hydroalcoholic fruit extract	Methyl gallate exhibited the strongest inhibitory action against *E. coli* and *P. aeruginosa* (1.25 mg/mL), while gallic acid demonstrated the lowest MIC against *S. typhi* (0.156 mg/mL), *L. monocytogenes*, and *S. aureus* (1.25 mg/mL). Gallic acid had the lowest MBC on *P. aeruginosa* and *L. monocytogenes*, while methyl gallate had the highest MBC on *P. aeruginosa* (2.50 mg/mL).	(24)
Gallic acid	Ethyl acetate fraction from hydroalcoholic fruit extract	The best MIC of Ac-FrCc was found against *A. hydrophila* (0.19 mg/mL), followed by *A. veronii* (0.39 mg/mL) and *A. dhakensis* (0.39 mg/mL). HECc demonstrated a lesser activity against *A. hydrophila* (1.56 mg/mL) and a higher potential for *A. veronii* and *A. dhakensis* (0.78 mg/mL). The highest MIC for Ac-FrEtCc was 0.09 mg/mL against *A. hydrophila*, followed by 0.78 mg/mL against *A. veronii* and *A. dhakensis*. The best MIC for gallic acid was 0.09 mg/mL against *A. hydrophila*, 3.12 mg/mL against *A. veronii*, and without activity against *A. dhakensis*.	(40)
Methanol extract	Leaves	Antibacterial activity on *S. aureus* (20 mg/mL), *Enterococcus faecalis* (290 mg/mL), and *P. aeruginosa* (270 mg/mL).	(89)
Silver nanoparticles synthesized using *C. coriaria* aqueous extract	Leaves	Antibacterial activity, observing a positive effect at 10 mg/mL on *E. coli* (6.66 mm), *P. aeruginosa* (13.6 mm), *K. pneumoniae* (10.0 mm), and *S. aureus* (6.66 mm).	(22)
0.5 g of dried fruit of *C. coriaria* mixed with sodium thioglycolate	Leaves	Antibacterial activity against *P. aeruginosa*, *K. pneumoniae*, and *Streptococcus pyogenes* except for *E. coli*.	(43)
Tannin fraction from acetone extract of pods	Pods	Antibacterial activity against *Salmonella typhimuriun*, *E. coli*, *P. aeruginosa*, Methicillin-resistant *S. aureus*, and *K. pneumonia*	(19)
Flavonoids and glycosides	Ethanolic extract from leaves	Carbohydrates reduced to 22.54–31.73 and 31–72 g/mL at zero and 24 h incubation of *Klebsiella pneumoniae* as per the cultures treated with glycoside and flavonoid compounds.	(41)

**Figure 3:**
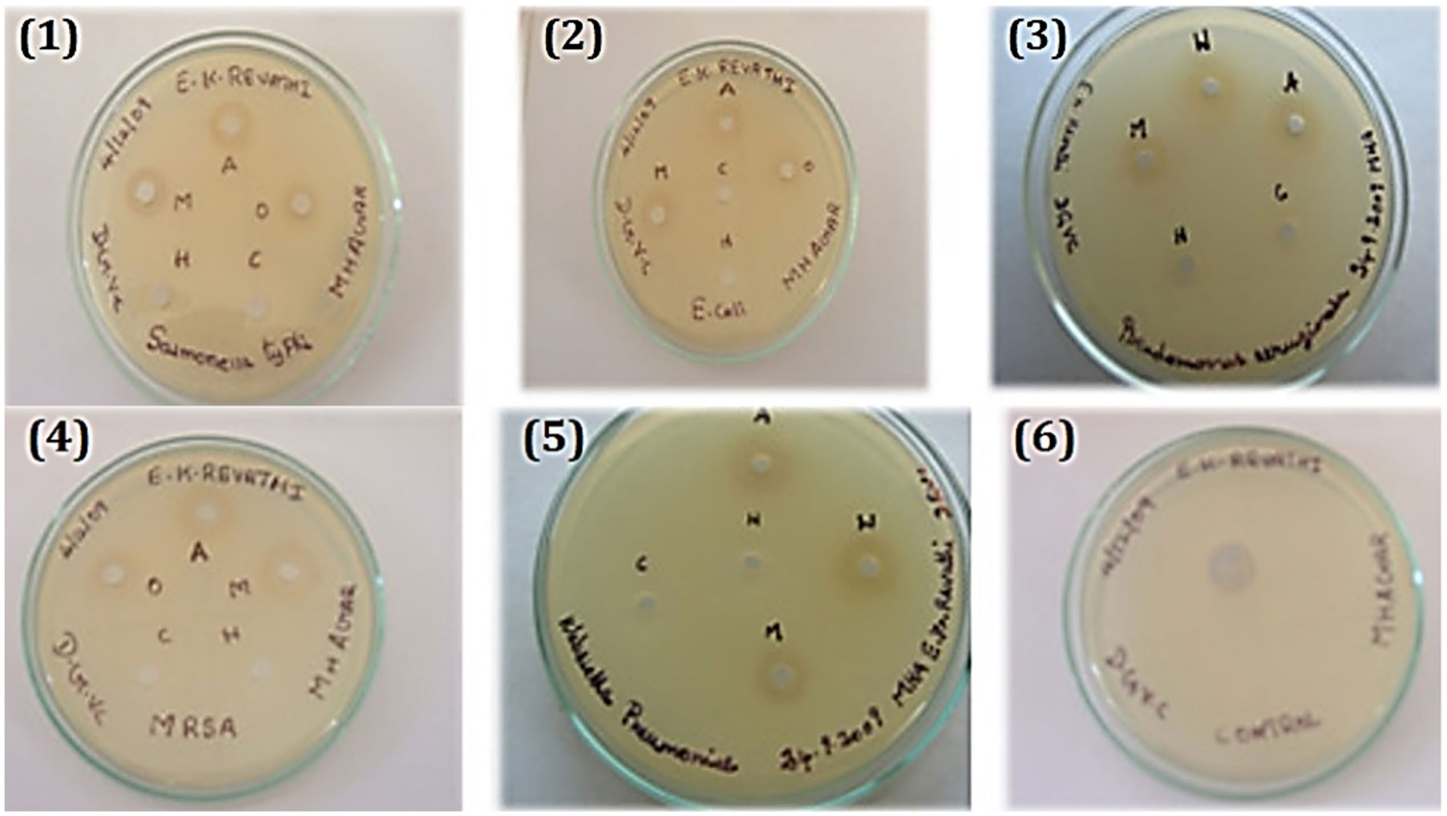
Antibacterial activity of bacterial strains toward *Caesalpinia coriaria* (19). 1. *Salmonella typhi*, 2. *Escherichia coli*, 3. *Pseudomonas aeruginosa*, 4. *Staphylococcus aureus*, 5. *Klebsiella pneumonia* and 6. Control. Reproduced from (19). This work is licensed under a Creative Commons Attribution 4.0 International License.

Treatment options for disorders caused by the genus *Aeromonas* may include the hydroalcoholic extract of *C. coriaria* and its fractions, which have antibacterial activity against *Aeromonas hydrophila*, *Aeromonas veronii*, and *Aeromonas dhakensis* (40). Ruminant parasitic nematodes were killed by the *C. coriaria* ethyl acetate fraction, with gallic acid serving as the primary chemical responsible for the observed ovicidal activity (15). *In vitro*, gallic acid also showed activity against bacteria of public health importance at concentrations of 5–10 mg/mL (24). Regarding the mechanism of action, the antibacterial activity of the methanolic extract of *C. coriaria* can be attributed to the presence of phenolic compounds, particularly phenolic acids; however, these are not the only secondary metabolites present in *C. coriaria* (18). It was reported that the antibacterial activity *in vitro* is attributed to the synergistic action of acidic and phenolic fractions, the activity of which is lost when these components are separated (42). Hexane, methanol, acetone, and water extracts by the maceration method from *C. coriaria* pod were tested *in vitro* against some pathogenic bacteria, and all of which are significantly inhibited by the acetone extract (19) as shown in Figure 3. Additionally, the plant extract was found to have an inhibitory activity of its acetone extract (10, 15, 20, 25, and 30 μg) on *S. typhi*, *E. coli*, *P. aeruginosa*, *S. aureus* and *K. pneumonia* (19). According to certain research, the fruit’s aqueous extract and the leaves’ alcoholic extract have antibacterial properties against *P. aeruginosa*, *E. coli*, *Xanthomonas pathovars*, and *S. aureus* (18, 43).

### Antiparasitic activity

One of the primary issues affecting small ruminants is parasitism, and among them, gastrointestinal nematodes (GIN) are the leading cause of death for sheep and goats in Mexico’s tropical regions (44–46). With a 70% prevalence in tropical areas, *Haemonchus contortus* is the most significant epidemiological nematode that parasitizes the abomasum of ruminants, including cattle, sheep, and goats (47, 48). The most dangerous parasite feeds on blood, damages the abomasal epithelium, and causes inflammation, emaciation, anemia, and hypoproteinemia in addition to submandibular edema, drooping of the productive parameters (the production of wool, milk, and meat), and frequently the death of infected animals (48, 49). From a biological and economic perspective, parasites are an issue, particularly when chemical dewormers are misused, which has aided in the emergence of anthelmintic resistance (50). One of the factors contributing to the development of resistance in these microorganisms is the regular use of anthelmintics to control parasites (51).

In this regard, medicinal plants are recommended to have bioactive compounds to act as nematicidal properties of use in ruminants (47, 52). Gallic acid is the chemical that caused the anthelmintic activity, as evidenced by the *in vitro* data, which demonstrated the larvicidal impact of the hydroalcoholic extract of *C. coriaria* against the nematode *H. contortus* (13). The most active effective concentrations with LC_50_ and LC_90_ values were 0.01 and 5.42 mg/mL, respectively. The animals in the *C. coriaria* fruit group did not exhibit a decrease in eggs per gram (EPG) at the beginning of the trial, which took place on day 7 of the first week. But by the end of the study (day 42), this group’s EPG reduction was much lower than that of the ivermectin-dewormed group (78.6 *vs* 52.6%) (13) for a group of animals that received the fruits of *C. coriaria* showed no reduction in the EPG. Under grazing conditions, this plant may act as a natural anthelmintic to prevent haemonchosis in goats. The study’s findings indicate that *C. coriaria* fruits have a significant anthelmintic effect in both *in vitro* and *in vivo* settings. The nematocidal qualities of the phytochemical elements derived from the fruits of this arboreal legume are demonstrated by the larvicidal impact (13).

Sheep naturally infected with mixed gastrointestinal nematode species that were fed 100 g of *C. coriaria* fruits showed a considerable reduction in eggs per gram of feces (70%) (9). Consequently, a notable anthelmintic impact in goats was attained at a 10% level of inclusion in the overall diet (53). Condensed tannins (CT) are abundant in the fruits of this arboreal legume; each gram contains 0.367 g of CT, according to Camacho-Díaz et al. (54). Recent research on the consumption of *C. coriaria* fruits (10% DM or 3.67% of CT) is comparable to that of Manuel-Pablo et al. (53), they determined that the levels of CT employed (1.5, 3.0, and 4.5%) in the basal diet did not affect productive parameters after evaluating the effect of *C. coriaria* fruit supplementation on goat productivity. The concentration affected the extracts’ inhibitory effects; for the acetonic extract, the inhibitory activity was very comparable to the positive control doses of 1.2 mg/mL, and for the ethanolic extract, 0.78 mg/mL (55). The results demonstrate that *H. contortus* eggs are inhibited by extracts prepared using *C. coriaria* fruits in acetonic and ethanolic solvents (55).

Hydroalcoholic extracts from *C. coriaria* leaves and mature fruits exhibit ovicidal effect on *H. contortus* and *H. placei* (Place, 1893) (Nematoda, Trichostrongylidae, Haemonchinae; 59). Hydro-alcoholic extracts from both leaves and fruits showed a concentration-dependent ovicidal activity, with a 25.0 mg/mL concentration showing 100% efficiency against both nematode species (56). The hatching suppression of five gastrointestinal parasitic nematode eggs (*Haemonchus* spp., *Cooperia* spp., *Ostertagia* spp., *Trichostrongylus* spp., and *Oesophagostomum* spp.) was linked to gallic acid and various galloyl derivatives extracted from *C. coriaria* fruits.

Hydro-alcoholic extract (HA-E) and ethyl acetate fraction (EtOAc-F) from the fruits showed ovicidal activity at an LC_50_ of 0.92 and 0.16 mg/mL, respectively (15). Galloyl derivatives showed ovicidal activity against cattle gastrointestinal parasitic nematodes close to 100% at a 1 mg/mL concentration. Additionally, it was demonstrated that methyl gallate was ineffective against these parasites (15).

### Possible bioactive mechanisms of the chemical compounds

Catechol, gallic acid, ethyl gallate, syringic acid, ferulic acid, caffeic acid, and cinnamic acid are members of the phenolic acid group, exhibiting diverse biological activities because of their antioxidant, anti-inflammatory, and antimicrobial properties (57). Catechol, a simple benzenediol, serves as a precursor in lignin and flavonoid biosynthesis, while gallic acid and its derivative ethyl gallate act as potent antioxidants with demonstrated antiparasitic activity (58). Hydroxycinnamic acids such as ferulic, caffeic, and cinnamic acid exhibited antibarasitoc effects on two *Haemonchus contortus* isolates (59). Ascorbic acid (vitamin C), pantothenic acid (vitamin B5), niacin (vitamin B3), and thiamine (vitamin B1) are essential water-soluble vitamins with critical metabolic functions (60). Ascorbic acid is a crucial antioxidant and cofactor in collagen synthesis, pantothenic acid is integral to coenzyme A synthesis and energy metabolism, niacin participates in redox reactions as NAD+/NADH, and thiamine (as thiamine pyrophosphate) is essential for carbohydrate metabolism and neurological function (61).

Catechin, luteolin, quercetin, chrysoeriol, kaempferol, rutin, naringin, and hesperidin are bioactive flavonoids, a diverse class of polyphenolic compounds widely found in plants and known for their significant antimicrobial properties (62). Catechin, a flavan-3-ol, shows strong antioxidant and cardioprotective effects, while luteolin and quercetin, both flavones and flavonols, respectively, exhibit potent antimicrobial and antiparasitic activities with ability to modulate signaling pathways and scavenge reactive oxygen species (63, 64). Chrysoeriol and kaempferol, also flavonols, contribute to plant defense mechanisms and display antimicrobial effects (65, 66). Naringin and hesperidin, classified as flavanone glycosides, are prevalent in citrus fruits, where they contribute to antioxidant defense, cholesterol metabolism, and anti-inflammatory responses, with hesperidin also enhancing cardiovascular health through the improvement of endothelial function (67, 68).

The natural compounds ellagic acid, valoneic acid, galloyl shikimic acid, 3-O-galloylquinic acid, digalloylquinic acid, digalloyl shikimic acid, and stigmasterol belong to distinct biochemical classes with significant biological and pharmacological importance (69, 70). Ellagic acid, a dimeric derivative of gallic acid, is a hydrolyzable tannin metabolite derived from ellagitannins and demonstrates potent antioxidant, anti-inflammatory, and anticancer properties by modulating cellular signaling pathways (71). The galloyl shikimic acid and digalloyl shikimic acid derivatives, intermediates in the shikimate pathway, are key precursors in plant polyphenol biosynthesis and exhibit antimicrobial and anti-inflammatory effects, particularly in *Eucalyptus* species as well as in Scots pine and Norway spruce (72, 73). Similarly, 3-O-galloylquinic acid and digalloylquinic acid, both classified as hydrolyzable tannins, are abundant in plants like *Terminalia chebula* and display hepatoprotective and antidiabetic activities through free radical scavenging and regulation of glucose metabolism (74). In contrast, stigmasterol, a phytosterol, is crucial for membrane structure in plants and exhibits cholesterol-lowering, anti-inflammatory, and immunomodulatory effects in humans, making it valuable in managing cardiovascular and metabolic diseases (75).

Monogalloylglucose, digalloylglucose, trigalloylglucose, tetragalloylglucose, and pentagalloylglucose are part of the class of hydrolyzable tannins, specifically gallotannins, which are esters of glucose and gallic acid with varying degrees of galloylation (76). These compounds show significant biological activities, including antioxidant, antimicrobial, and anti-inflammatory properties, because of their capacity to scavenge free radicals and interact with cellular proteins and enzymes (77). Galloyltannins, a broader subgroup, demonstrate enhanced bioactivity with increasing galloyl units, as observed in pentagalloylglucose, which is particularly noted for its potent protein-binding and enzyme-inhibitory effects (78). Hexahydroxydiphenyl-digalloylglucose, an ellagitannin precursor, further adds to the structural diversity and functional importance of tannins, contributing to potential therapeutic applications (21).

Most of the studies about the chemical compounds identified in *C. coriaria* showed the presence of polyphenolic compounds including gallic acid, galloyl derivatives, tannins, and flavonoids. From other plant species, the methanol extracts of *Ceratonia siliqua* L. (family Fabaceae) and *Ziziphus spina-christi* (L.) Desf. (family Rhamnaceae) leaves and branched showed the presence of several bioactive compounds including gallic acid, syringic acid, methyl gallate, catechin, coumaric acid, ellagic acid, and chlorogenic acid with potential antifungal activities (27).

The biological activity of flavonoids seems to be due to their amphipathic characteristics, which strengthen the chemical structure’s antibacterial activities, especially those of the hydrophobic substituents (prenyl groups, alkylamino, and alkyl chains, and heterocyclic fractions with N or O) (79, 80). Quercetin, rutin, vanillic acid, caffeic acid, apigenin, chlorogenic acid, ferulic acid, and cinnamic acid have been reported in polar extracts of various medicinal plants (81, 82).

In many ways, gallic acid is one of the most significant plant polyphenols with health-promoting properties. Numerous investigations have demonstrated that gallic acid suppresses bacterial growth by changing the shape of the membrane, bacterial metabolism, and the development and growth of biofilms (83). Disrupting bacterial cell membranes, preventing the formation of biofilms, and possibly interfering with bacterial DNA repair pathways are the main ways that gallic acid works against bacteria. Additionally, it can make other antibiotics more effective (84, 85). *E. coli*, *P. aeruginosa*, *S. aureus*, and *L. monocytogenes* were used to test the mechanisms of action of gallic and ferulic acids, hydroxybenzoic acid, and hydroxycinnamic acid. Through changes in hydrophobicity, a decrease in negative surface charge, and the occurrence of local rupture or pore formation in the cell membranes with the subsequent leakage of vital intracellular constituents, gallic and ferulic acids caused irreversible changes in membrane properties (charge, intra and extracellular permeability, and physicochemical properties) (86, 87).

## Conclusions and future perspectives

*Caesalpinia coriaria* is shown as an alternative for the formulation of antimicrobial and anthelmintic drugs due to its content of bioactive compounds. *C. coriaria* is a promising source of bioactive molecules with various applications, including anthelmintic properties, antibacterial effects, and environmental benefits. From the future perspective, *in vivo* studies or concrete industrial applications should be done, in order to provide greater practical value to the extracts from several botanical parts of *C. coriaria*, which are rich in phenolic and tannin chemicals, have the potential to improve agriculture, medicine, and sustainable technologies.
